# SELIMETRY—a multicentre I-131 dosimetry trial: a clinical perspective

**DOI:** 10.1259/bjr.20160637

**Published:** 2017-05

**Authors:** Jonathan Wadsley, Rebecca Gregory, Glenn Flux, Kate Newbold, Yong Du, Laura Moss, Andrew Hall, Louise Flanagan, Sarah R Brown

**Affiliations:** ^1^ Weston Park Hospital, Sheffield, UK; ^2^ Department of Physics, The Royal Marsden NHS Foundation Trust and Institute of Cancer Research, Sutton, UK; ^3^ Royal Marsden Hospital, London, UK; ^4^ Velindre Hospital, Cardiff, Wales; ^5^ CTRU, University of Leeds, Leeds, UK

## Abstract

Treatment options for patients with thyroid cancer that is no longer sensitive to iodine therapy are limited. Those treatments which currently exist are associated with significant toxicity. The SELIMETRY trial (EudraCT No 2015-002269-47) aims to investigate the role of the MEK inhibitor Selumetinib in resensitizing advanced iodine refractory differentiated thyroid cancer to radioiodine therapy. Patients deemed to have sufficient iodine uptake in previously iodine refractory lesions after 4 weeks of Selumetinib therapy will be given an empirical activity of 5.5 GBq I-131, and response to therapy will be assessed. The trial presents an opportunity to investigate the dosimetric aspects of radioiodine therapy for advanced thyroid cancer. Patients will undergo serial I-123 single-photon emission CT (SPECT)/CT scans following Selumetinib therapy to determine whether there has been a change in the degree of iodine uptake to justify further I-131 therapy, and to allow dosimetric calculations to predict absorbed dose to target lesions following therapy. Patients receiving I-131 therapy will undergo a further series of post-therapy SPECT/CT scans to allow dosimetric calculations. We describe the challenges in setting up a multicentre trial in a relatively underinvestigated field, describing the work that has been carried out to calibrate and validate measurements to ensure that standardized image data are collected at each site. We hope that this trial will lead to individualization and optimization of therapy for patients with advanced thyroid cancer and that the ground work carried out in setting up a network of centres capable of standardized molecular radiotherapy dosimetry will lead to further clinical trials in this field.

## THE SELIMETRY TRIAL

Differentiated thyroid cancer is the most common endocrine malignancy, with 3200 patients diagnosed in the UK in 2012.^1^ Most patients, presenting with early disease, are cured by surgical resection of part of or the whole thyroid. In selected cases, this is followed by radioiodine ablation therapy. Around 5–10% of patients will develop more advanced disease not amenable to complete surgical resection.

I-131 is an important treatment for advanced differentiated thyroid cancer, proving curative in a proportion of patients and controlling the disease for several years in many more. Unfortunately, a significant number of cancers will not respond to I-131 in the first instance or become resistant following a number of I-131 therapies. This is described as iodine refractory differentiated thyroid cancer (RR-DTC). Currently, treatment options for this group of patients are limited.

The SELIMETRY trial (EudraCT No 2015-002269-47) is a multicentre Phase 2 trial investigating the potential for Selumetinib to resensitize RR-DTC cells to radioiodine therapy. Selumetinib is an oral agent that selectively inhibits MEK, a target in the MAPK signalling pathway. Aberrations in this pathway are known to be associated with loss of uptake of iodine by thyroid cancer cells. Laboratory work and a pilot study in humans have shown promising results.^2^ In the pilot study, 20 patients with advanced RR-DTC were treated with Selumetinib 75 mg twice a day for 28 days. Patients underwent an I-124 positron emission tomography (PET) scan before and after treatment. Those patients predicted to achieve a lesional dose of 20 Gy or greater on the basis of the post-treatment I-124 PET scan went on to receive further I-131 therapy. 8 (40%) of the 20 patients went on to receive I-131 therapy, all with evidence of either radiological response or disease stabilization.

The SELIMETRY trial aims to replicate these findings in a multicentre setting, with a larger cohort of 60 patients, to demonstrate the ability of Selumetinib and further I-131 to prolong progression-free survival, and to further investigate and develop the dosimetric aspects of the treatment.

## I-131 DOSIMETRY

I-131 has been used as an effective treatment for thyroid cancer since the 1940s. It is used both in the adjuvant setting, to reduce the risk of recurrence after surgery, and as a treatment for metastatic disease. Current UK guidelines recommend the use of empirical activities of I-131, since “the role of dosimetry and its impact on clinical outcomes compared to empirical use of I-131 therapy is unclear”.^3^

However, the use of an empirical activity for therapy is known to lead to a very wide variation in absorbed dose delivered to the target. One study investigating absorbed dose to thyroid remnant showed that for a fixed activity administration, absorbed dose to the thyroid remnant varied from 1.2 Gy to 540 Gy.^4^ Since response to therapy is very likely to be determined by absorbed dose delivered to the target, it is critical that we have a clearer understanding of the dose delivered both to tumour and to organs at risk. This has led to calls for further development of dosimetry techniques for molecular radiotherapy.^5,6^

In the SELIMETRY trial, patients found to have increased iodine uptake on post-Selumetinib imaging will undergo a series of 3–4 single-photon emission CT (SPECT)/CT scans (Table 1), to allow calculation of predicted absorbed dose to each lesion identified when I-131 therapy is administered.

**Table 1.** Patient scanning schedule

Baseline I-123 scan (under rhTSH stimulation)	
24 ± 4 h (post-I-123)	WB and SPECT/CT scan
4 weeks on Selumetinib	
Post-Selumetinib 4× I-123 scans (under rhTSH)	
5 ± 1 h (post-I-123)	WB and SPECT/CT scan
24 ± 4 h (post-I-123) to be compared with the baseline scan	WB and SPECT/CT scan (match the baseline time between injection and scanning)
48 ± 4 h (post-I-123)	SPECT/CT
72 ± 4 h (post-I-123)	SPECT/CT
Iodine-131 therapy administration (5.5 GBq) (under rhTSH stimulation) and dosimetry scans	
24 ± 4 h (post-I-131) dead-time permitting	WB and SPECT/CT scan
48 ± 4 h (post-I-131)	SPECT/CT
72 ± 4 h (post-I-131)	SPECT/CT
144 ± 24 h (post-I-131)	SPECT/CT

rhTSH, recombinant human thyroid stimulating hormone; SPECT, single-photon emission CT; WB, Whole Body.

In the absence of any evidence to support a dose threshold required for effective therapy, all patients judged to have increased iodine uptake on post-Selumetinib imaging at central review will go on to receive an empirical activity of 5.5 GBq I-131 therapy. Patients without increased uptake will continue to be treated as per local practice. Three or four further SPECT/CT scans (Table 1) will be taken post-therapy to determine that actual absorbed dose to identified lesions.

Patients will then be followed up clinically and with regular imaging to determine the radiological and biochemical response rate, as determined by changes in the thyroid-specific tumour marker, thyroglobulin. This will allow the exploration of the relationship between response rate and absorbed dose, to assess whether there may be a threshold absorbed dose level necessary for response.

In addition, whole-body count readings will be made throughout the patient hospital stay to calculate their whole-body dose. This dose may correlate with myelotoxicity and therefore could be used to calculate the maximum tolerated activity that could be administered to the patient.

## CHALLENGES

### Historical barriers to dosimetry

Molecular radiotherapy plays an increasingly important role in the treatment of a number of malignancies. Although outcome for any given patient is dependent on the absorbed doses delivered rather than on activity administered, dosimetry has not been implemented routinely as is mandatory for external beam radiotherapy. A number of reasons have led to this anomaly, stemming from a lack of reimbursement and a lack of legal requirements for imaging or dosimetry calculations.

There are a number of significant differences in the radiation delivery from molecular radiotherapy when compared with external beam radiotherapy. Radionuclides deliver a continuous, variable low-dose rate radiation to the tumour, and this dose rate is patient dependent. It is affected by multiple factors including the perfusion of the tumour, the affinity of the vector, the cellular washout rate and the half-life of the radionuclide. It increases during the uptake phase and decreases subsequently, necessitating determination of a time–activity curve.

To date, there have been no multicentre trials to investigate dose–effect correlations in radioiodine treatment of thyroid cancer, although there is increasing evidence of such correlations.^7^ Current guidelines recommend the administration of empirical activity of I-131 in the absence of any evidence base for dosimetry-based prescribing.

### Scanning facilities

It had initially been hoped to use I-124 PET imaging pre- and post-Selumetinib therapy, as in the pilot study reported above, as this allows higher resolution imaging and potentially more accurate predictive dosimetry. However, there is currently no commercial supplier of I-124, and many UK centres have limited access to PET scanning facilities using tracers other than fludeoxyglucose. A pragmatic decision was therefore taken to use I-123 SPECT/CT, which is widely available in UK centres and should give adequate information to guide treatment decisions.^8^

### Funding

A significant investment of time has been required to calibrate and validate SPECT/CT measurements at each site to ensure consistent dosimetry and permit multicentre data interpretation. The requirement for serial SPECT/CT scans is demanding of scanner time and expensive, but necessary to allow adequate dosimetry calculations. The work has been generously supported by a CRUK grant, Astrazeneca, who have provided the Selumetinib, and a further per-patient grant to support the study, and Genzyme, who have supplied recombinant TSH for use before scans and therapy.

It is hoped that once a network of centres capable of standardized dosimetry is established, the costs of supporting future trials will be reduced.

### Standardization

It is clearly critical that image data collected at each study site is optimized and standardized to allow accurate dosimetry calculations. Early in the trial development, a meeting of medical physicists from each of the participating centres, along with a representative from National Physical Laboratory and the National Cancer Research Institute Radiotherapy Trials Quality Assurance group, was convened to discuss scanning protocols and ensure that protocol requirements could be met at every site. This meeting proved invaluable, allowing sharing of best practice and development of a workable protocol.

Subsequently, each site has been visited prior to study opening to calibrate and validate SPECT/CT measurements. Prior to the visit, centres are required to perform routine quality control measurements and to ensure that their SPECT/CT system is set up with the appropriate parameters (Table 2).

**Table 2.** Scanning parameters

Parameter	I-123	I-131
Collimator	MEGP	HEGP
Peak energy window (20%)	159 keV (138.9–180.3 keV)	364 keV(327.6–400.5 keV)
Low scatter energy window (6%)	67.4–138.9 keV	308.5–327.6 keV
High scatter energy window (6%)	180.3–348.9 keV	400.6–425.4 keV
Matrix	128 × 128	
SPECT movement	Body contour (or radius as close to the phantom as possible)	
Projections	72 (5°/projection)	
Time per projection	60 s	
CT	Standard low-dose protocol	

HEGP, high-energy general purpose; MEGP, medium-energy general purpose; SPECT, single-photon emission CT.

During the visit, I-131 dead-time is characterized based on count rate measurements for increased activity (up to 2.8 GBq) in a cylindrical (20 cm diameter, 20 cm long) phantom. I-131 and I-123 calibration factors to correct partial volume and resolution effects are also measured. These are determined from SPECT/CT images of known activity concentrations of I-131 or I-123 in cylindrical inserts, with volumes from 0.5 ml to 196 ml, positioned in a non-active water-filled anthropomorphic phantom.

To ensure that consistent decisions are made about which patients should go on to receive I-131 following Selumetinib, it has been decided that post-Selumetinib SPECT/CT scans will be centrally reviewed in real time. Whilst logistically challenging, this is felt to be important to ensure the credibility of the final results. With the use of electronic transfer of imaging data, we believe that this will be feasible within the necessary timeframes.

## SUMMARY

The SELIMETRY trial presents a unique opportunity to develop the infrastructure and expertise necessary in UK nuclear medicine centres to allow I-131 dosimetry calculations to be performed so that absorbed dose to target lesions can be reported in a consistent and reproducible manner.

It is hoped that this study will lead towards defining a dose threshold for successful treatment of advanced thyroid cancer and to individualizing administered activity for future patients to ensure that this dose threshold is reached whilst minimizing toxicity.

It is also hoped that having developed a network of centres capable of standardized dosimetry, further clinical trial protocols will be developed to establish an evidence base for dosimetry-guided molecular radiotherapy.

## FUNDING

The SELIMETRY trial is supported by a Cancer Research UK Grant. Astra Zeneca have provided the study drug and a per-patient fee. Sanofi Genzyme have supplied recombinant TSH free of charge for the study. GE have provided a discount for I-123 used in the study. Butterfly Thyroid Cancer Trust are providing support for the protein bound iodine substudy.

## References

[1] CRUK: [cited 2016 May]. Available from: http://www.cancerresearchuk.org/health-professional/cancer-statistics/statistics-by-cancer-type/thyroid-cancer

[2] Ho AL, Grewal RK, Leboeuf R, Sherman EJ, Pfister DG, Deandreis D (2013). Selumetinib-enhanced radioiodine uptake in advanced thyroid cancer. *N Engl J Med*.

[3] Perros P, Boelaert K, Colley S, Evans C, Evans RM, Gerrard BG (2014). Guidelines for the management of thyroid cancer. *Clin Endocrinol (Oxf)*.

[4] Sgouros G, Kolbert KS, Sheikh A, Pentlow KS, Mun EF, Barth A (2004). Patient-specific dosimetry for 131I thyroid cancer therapy using 124I PET and 3-dimensional-internal dosimetry (3D-ID) software. *J Nucl Med*.

[5] McGowan DR, Guy MJ (2015). Time to demand dosimetry for molecular radiotherapy?. *Br J Radiol*.

[6] CTRad 2016. Available from: http://www.ncri.org.uk/wp-content/uploads/2016/06/CTRad-promoting-research-in-MRT-UK-June-2016.pdf

[7] Flux GD, Haq M, Chittenden SJ, Buckley S, Hindorf C, Newbold K (2012). A dose-effect correlation for radioiodine ablation in differentiated thyroid cancer. *Eur J Nucl Med Mol Imaging*.

[8] Canzi C, Zito F, Voltini F, Reschini E, Gerundini P (2006). Verification of the agreement of two dosimetric methods with radioiodine therapy in hyperthyroid patients. *Med Phys*.

